# The Imperative of Public Health Expertise in Ecuadorian Health Leadership: A Call for Competency-Based Appointments

**DOI:** 10.3389/ijph.2024.1607894

**Published:** 2025-01-07

**Authors:** Esteban Ortiz-Prado, Isaac Alexander Suárez Sangucho, W. Ricardo Cañizares Fuentes, Jorge Vasconez-Gonzalez, Juan S. Izquierdo-Condoy

**Affiliations:** ^1^ One Health Research Group, Universidad de Las Américas, Quito, Ecuador; ^2^ Universidad Católica de Santiago de Guayaquil UCSG y Universidad de Guayaquil UG, Guayaquil, Ecuador

**Keywords:** public health leadership, health policy, health system, infant mortality, Ecuador

## Commentary Letter

In the past three decades, Ecuador’s health system has faced frequent ministerial turnover, often appointing leaders with limited training in public health policy and management. Although many ministers have strong clinical backgrounds, their lack of public health expertise has hindered a cohesive vision, raising concerns about the leadership’s capacity to tackle the nation’s complex health challenges effectively. The Ministry of Health is responsible for upholding the right to health, promoting health, preventing diseases, overseeing surveillance, and providing integrated care. It also develops clinical protocols and management guidelines, delivers health services, and conducts studies comparing national and international health management best practices [1, 2]. To adequately fulfill these responsibilities, a master’s degree in public health should be the minimum requirement for those considered for the position of Minister of Health. While ministers are indeed supported by technical personnel, they must have enough training to understand technical matters and not be overly reliant on staff or influenced by conflicts of interest [3]. Advanced public health training equips leaders with essential knowledge about financing, management, cost-effectiveness, and health communication, enabling them to evaluate and implement comprehensive health policies [4, 5].

A health minister requires both managerial skills to oversee health services and advocacy skills to ensure that potential effects on population health are integrated into the work of other government departments and ministries [6]. Helath Ministers require strong knowledge of epidemiology and public health preparedness, highlighted by challenges from global health crises like COVID-19. However, the Ministry faces internal issues impacting ministerial effectiveness, including overlapping responsibilities in regulations, unclear accountability, fragmented technical programs, and departments prioritizing specific professions over broader functions [7]. Furthermore, governance within the health sector is heavily influenced by institutional power dynamics. This lack of governance can severely hinder the performance of the Ministry of Health, contributing to systemic failures [8, 9].

Over the past 30 years in Ecuador, most health ministers have lacked advanced degrees or experience in key areas such as public health, epidemiology, or health systems management (see Table 1). This absence of qualifications hinders effective public health leadership, which demands an integrated, evidence-based approach to address social, environmental, and behavioral health determinants [10–13].

**TABLE 1:** Description of ministers of the Ministry of Public Health of Ecuador during the 21st century.

Name	Gender	Age at the time of assuming the position	Period		Education up to the date of assuming the position of minister of health	Program o project	Appointing government
Edgar Rodas Andrade	Male	62 years	August 10, 1998	January 21, 2000	• Doctor of Medicine and Surgery	The Cotacachi experience of decentralization and the formation of a health council was developed	Jamil Mahuad
Fernando Rodrigo Bustamante Riofrío	Male	N/A	January 22, 2000	January 23, 2001	• Doctor of Medicine and Surgery • Specialist in Internal Medicine^a^		Gustavo Noboa
Patricio Jamriska Jácome	Male	N/A	January 23, 2001	October 1, 2002	• Doctor of Medicine and Surgery • Specialist in Gynecology and Obstetrics^a^		
Vicente Antonio Habze Auad	Male	N/A	October 1, 2002	January 15, 2003	• Doctor of Medicine and Surgery		
Francisco Xavier Andino Rodríguez	Male	38 years	January 15, 2003	August 22, 2003	• Doctor of Medicine and Surgery • Master’s Degree in Epidemiology^a^ • Specialist in Neurology^a^	The pentavalent vaccine was introduced into the vaccination schedule. The strategy against malaria was changed to early diagnosis and timely treatment. Treatment of HIV-AIDS with antiretrovirals was started and the program was strengthened together with that of sexually transmitted diseases	Lucio Gutiérrez
Ernesto Macario Gutiérrez Vera	Male	65 years	August 22, 2003	December 17, 2003	• Doctor of Medicine and Surgery • Specialist in Clinical Pathology^a^		
Teófilo Lama Pico	Male	66 years	December 17, 2003	April 20, 2005	• Doctor of Medicine and Surgery	Strengthening of health areas. Development of Universal Health Insurance (AUS). Beginning of the process of decentralization of health to municipalities (Quito, Guayaquil, Cotacachi)	
Wellington Sandoval Córdova	Male	66 years	April 22, 2005	December 29, 2005	• Doctor of Medicine and Surgery		Alfredo Palacio
Iván Jacinto Zambrano Cedeño	Male	N/A	December 29, 2005	May 30, 2006	• Doctor of Medicine and Surgery		
Guillermo José Wagner Cevallos	Male	N/A	May 30, 2006	January 15, 2007	• Doctor of Medicine and Surgery • Higher Diploma of Fourth Level in Local Development and Health • Specialist in Gynecology and Obstetrics^a^		
Caroline Judith Chang Campos	Female	46 years	January 15, 2007	April 21, 2010	• Doctor of Medicine and Surgery • Specialist in Health Service Management • Master’s degree in health management for Local Development^a^ • Doctorate in Health Sciences^a^	Implementation of the transformation process of the health sector. Tariff for the exchange of services in the public and complementary network. New Constitution that incorporates health as a right and universal access. Health system based on primary healthcare. Accelerated plan to reduce maternal mortality. Development of the Comprehensive Family and Community Healthcare Model and implementation of basic health teams. 8 new vaccines were incorporated into the Expanded Immunization Program	Rafael Correa
David Chiriboga Allnutt	Male	N/A	April 21, 2010	January 13, 2012	• Doctor in Medicine and Surgery		
Carina Isabel Vance Mafla	Female	35 years	August 23, 2012	November 13, 2015	• Bachelor of History and Political Science • Master of Public Health	Implement labeling of processed foods	
Margarita Beatriz Guevara Alvarado	Female	N/A	November 13, 2015	January 6, 2017	• Doctor of Medicine and Surgery • Master of Sexual Education^a^		
María Verónica Espinosa Serrano	Female	34 years	January 6, 2017	May 24, 2017	• Medical Doctor • Master of Public Health^a^	Implementation of a medical program in the neighborhood	
			May 24, 2017	July 3, 2019			Lenin Moreno
Catalina de Lourdes Andramuño Zeballos	Female	N/A	July 3, 2019	March 21, 2020	• Doctor of Medicine and Surgery • Specialist in Health Service Management • Master of in Public Health • Doctorate in Public Management and Governance^a^		
Juan Carlos Zevallos López	Male	63 years	March 21, 2020	March 1, 2021	• Doctor of Medicine and Surgery • Specialization in Cardiology		
Rodolfo Enrique Farfán Jaime	Male	63 years	March 1, 2021	March 19, 2021	• Doctor of Medicine and Surgery • Higher Diploma in Higher Education • Specialist in General Surgery • Master’s Degree in higher Education • Doctor of Education^a^		
Mauro Antonio Falconí García	Male	45 years	March 19, 2021	April 8, 2021	• Doctor of Medicine and Surgery • Specialist in Emergency Medicine and Disasters		
Camilo Aurelio Salinas Ochoa	Male	38 years	April 8, 2021	May 24, 2021	• Doctor of Medicine and Surgery • Master’s degree in health management and administration		
Ximena Patricia Garzón Villalba	Female	51 years	May 24, 2021	July 7, 2022	• Doctor of Medicine and Surgery • Doctor of Philosophy Public Health Occupational Health for Health Professionals	Implemented the vaccination against COVID-19 with the coordination of various sectors. Before a ministerial program was a presidential program	Guillermo Lasso
José Leonardo Ruales Estupiñan	Male	65 years	July 7, 2022	November 23, 2023	• Doctor of Medicine and Surgery • Specialist in Health Research and Administration	The 10-year health plan was drawn up	
Franklin Edmundo Encalada Calero	Male	49 years	November 23, 2023	1 June 14, 2024	• Doctor of Medicine • Specialist in General Surgery • Master’s Degree in Curriculum Design • Master’s Degree in Health Management and Direction		Daniel Noboa
Manuel Antonio Naranjo Paz y Miño	Male	69 Years	June 18, 2024	Current	• Doctor of Medicine • Specialist in Internal Medicine		

^a^ Studied during or completed after the MoH appointment.

**FIGURE 1:**
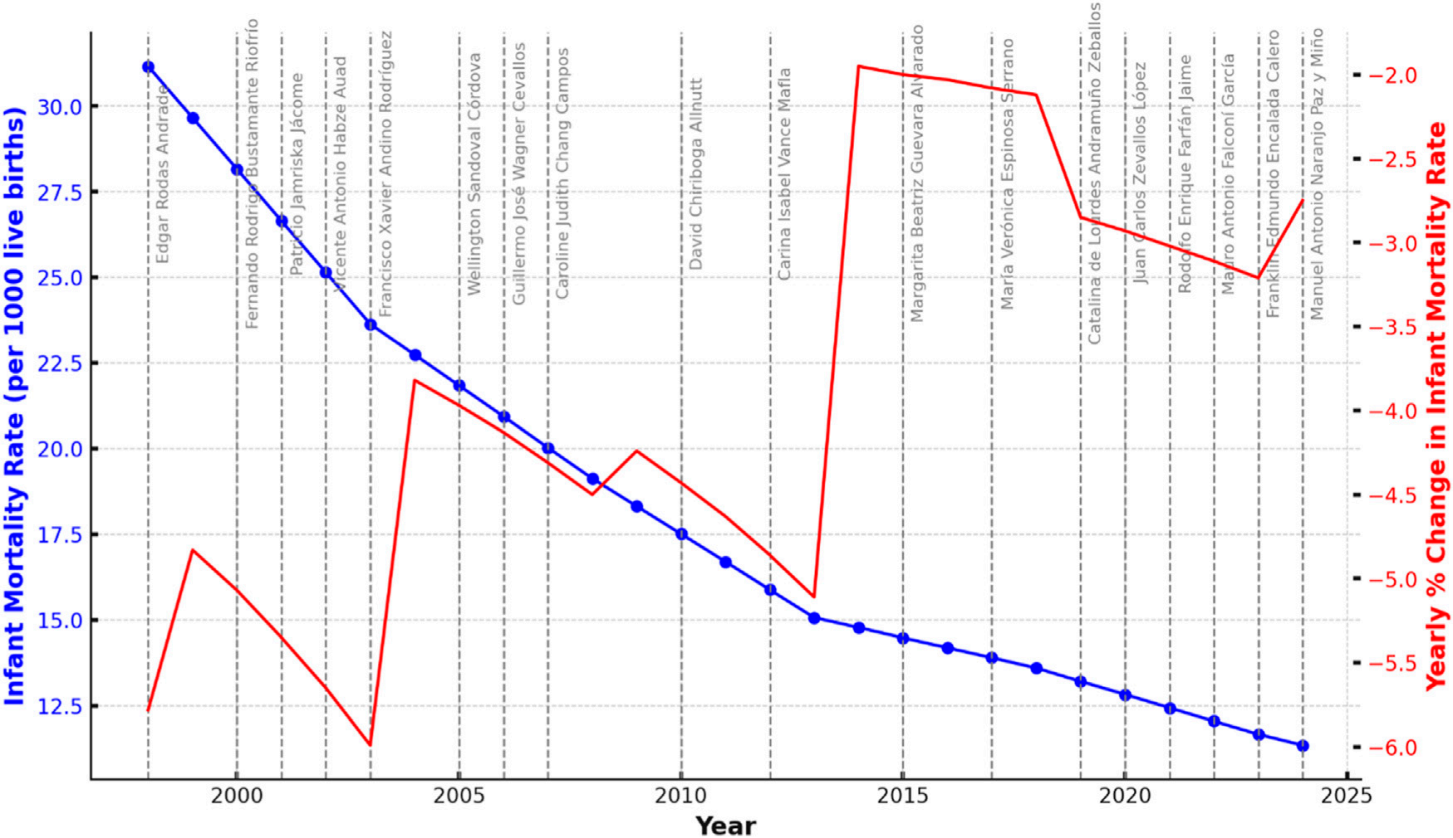
Trends in Infant Mortality Rate in Ecuador (1998–2024) with Ministerial Terms Highlighted. The blue line represents the infant mortality rate per 1,000 live births, while the red line shows the yearly percentage change in the rate. Gray vertical lines indicate changes in ministerial leadership, with ministers labeled.

Although the primary role of a health minister is to manage and oversee the national health system, their responsibility also extends to proposing public policies that can be evaluated through tangible improvements in health indicators, such as infant mortality. We believe that this is a significant issue for health governance. Therefore, we conducted an analysis based on the significant reductions in infant mortality during specific periods, identifying the ministers in charge and the outcomes associated with their tenure, highlighting changes that could reflect the impact of effective leadership and policy implementation (Figure 1).

Although not much can be inferred from non-causal ecological data, the graph illustrates Ecuador’s infant mortality rate (IMR) from 1998 to 2024, with percentage changes between consecutive years in red. It shows some notable changes. Ministerial changes are marked by vertical dashed lines, with ministers identified by name. There has been a consistent decline in IMR from 31.17 deaths per 1,000 live births in 1998 to 11.345 in 2024, although the rate of decline has slowed in recent years. The most significant decreases occurred between 2000–2005 and 2009–2012. Although the reduction in infant mortality is influenced by many circumstances and determinants typical of a country transitioning from low to middle income, a noticeable slowdown in the decline of the infant mortality rate (IMR) occurred between 2010 and 2020. More recently, (2021–2023) oversaw modest declines in the IMR, despite the challenges posed by the COVID-19 pandemic (Table 2).

**TABLE 2:** Ecuadorian ministers of health (1998–2024), infant mortality rates (IMR), and total percentage change in IMR by ministerial tenure.

Minister	Period	Start IMR	End IMR	Total change (%)	MPH degree
Edgar Rodas Andrade	1998–2000	31.170	28.159	**−9.65%**	No
Fernando Rodrigo Bustamante Riofrío	2000–2001	28.159	26.653	**−5.35%**	No
Patricio Jamriska Jácome	2001–2002	26.653	25.148	**−5.64%**	No
Vicente Antonio Habze Auad	2002–2003	25.148	23.642	**−5.99%**	No
Francisco Xavier Andino Rodríguez	2003–2005	23.642	21.837	**−7.64%**	Yes (MPH)
Wellington Sandoval Córdova	2005–2006	21.837	20.935	**−4.13%**	No
Guillermo José Wagner Cevallos	2006–2007	20.935	20.032	**−4.32%**	No
Caroline Judith Chang Campos	2007–2010	20.032	17.507	**−12.60%**	Yes (MPH)
David Chiriboga Allnutt	2010–2012	17.507	15.884	**−9.28%**	No
Carina Isabel Vance Mafla	2012–2015	15.884	14.484	**−8.82%**	Yes (MPH)
Margarita Beatriz Guevara Alvarado	2015–2017	14.484	13.895	**−4.07%**	Yes (MPH)
María Verónica Espinosa Serrano	2017–2019	13.895	13.214	**−4.91%**	Yes (MPH)
Catalina de Lourdes Andramuño	2019–2020	13.214	12.827	**−2.93%**	Yes (MPH)
Juan Carlos Zevallos López	2020–2021	12.827	12.440	**−3.02%**	No
Ximena Patricia Garzón Villalba	2021–2022	12.440	12.053	**−3.11%**	Yes (MPH)
Franklin Edmundo Encalada Calero	2023–2024	11.666	11.345	**−2.75%**	No
Manuel Antonio Naranjo Paz y Miño	2024-on	11.345	N/A	**N/A**	No

Bold values in the table represent the largest percentage decrease in Infant Mortality Rate (IMR) during the tenure of a specific Minister of Health, highlighting periods of the most significant improvements in public health outcomes.

In Ecuador, significant milestones in public health were achieved, particularly before the year 2000. Key figures during their tenure led important vaccination campaigns and successfully managed the 1991 cholera outbreak, reducing infant mortality and advancing the Comprehensive Family and Community Health Program [14]. Between 1990 and 2006, the country saw critical reforms, such as the decentralization of health management to municipalities, the formation of Cantonal Health Councils, and the proposal of Universal Health Insurance in 2005–2006 [15]. However, in 2009, these decentralization efforts were reversed with the re-centralization of the health management system [16], undoing much of the progress that had been made.

During 2014, the introduction of new food labeling regulations, which made nutritional information more accessible and positioned Ecuador as a global reference in non-communicable disease prevention was an important contrbution. However, this period was also marked by controversial decisions, including the closure of the National Institute of Hygiene and Tropical Medicine, the elimination of the National Service for the Eradication of Malaria (SNEM), and the shutdown of vaccine production in Ecuador [17]. Despite these public health initiatives, the reduction in infant mortality rates (IMR) during this period was less pronounced compared to other periods. While some improvements were made, the IMR did not decrease as significantly as might have been expected, highlighting a period where public health outcomes did not fully align with the scale of reforms introduced.

The COVID-19 pandemic highlighted Ecuador’s severe shortage of public health expertise, resulting in one of the world’s highest excess death rates. Health ministers, often lacking local experience, communication skills, and disease management knowledge, struggled to provide clear public health messaging. This was worsened by significant mismanagement and corruption, including inflated prices for essential medications and supplies, which deepened the crisis [18, 19].

Corruption has long plagued the health sector, with some ministers facing serious allegations. For example, several scandals have involved the procurement of ambulances and other essential supplies at inflated prices, breaching public procurement laws [20–22].

Ecuador’s health leadership has historically been marked by high ministerial turnover, driven by political interests. This instability, coupled with instances of corruption and controversial policies, has hindered effective public health initiatives and created fragmented health policies. While some achievements exist, persistent leadership issues have led to high malnutrition rates and ineffective campaigns on issues like traffic accidents and drug abuse, contrasting sharply with the successes of neighboring countries like Peru.

Appointing leaders focused solely on clinical medicine without a robust public health background poses several risks:

• **Fragmented Policies**: Lacking public health foundations can lead to ineffective, fragmented policies [13, 23, 24].
• **Curative Bias**: Overreliance on treatment instead of prevention perpetuates unsustainable healthcare costs [25].
• **Insufficient Emergency Preparedness**: COVID-19 underscored the need for leaders skilled in epidemiology and crisis management [26–28].

To better manage Ecuador’s health system, technical skills and public health experience should be prioritized over political considerations in minister selection. Ideal candidates would possess:

• Advanced public health qualifications.
• Proven experience in public health policy formulation and evaluation.
• Active public health research engagement.
• Strong leadership and communication skills to articulate a public health vision and make informed, evidence-based decisions.

Recent ministers have lacked communication competencies, resulting in fewer public health campaigns and diminishing the perception of health ministers as public health advocates.

Selecting health ministers is a nuanced task influenced by social, political, and contextual variables, particularly in developing nations like Ecuador. However, the logic and some of the evidence underscores that this process must be approached thoughtfully, prioritizing technical expertise over political considerations, to ensure sustainable public health progress [29–33]. While the public often expects a Minister of Health to be an effective administrator, adept at managing public procurement and addressing operational challenges, what Ecuador urgently requires is a leader with expertise in prevention, health promotion, and ensuring equitable access to healthcare services.

These competencies are often lacking in physicians focused on curative, private-sector roles. This commentary highlights systemic issues, not as a complaint but as a reflection on persistent shortcomings. For example, despite a 25-year national malnutrition prevention program, Ecuador still has one of the region’s highest malnutrition rates. Even during economic booms, investment favored hospital infrastructure over essential primary healthcare. This manuscript urges Ecuadorian authorities to address these issues and adopt the recommended steps for strengthening national health leadership, shifting towards a comprehensive public health focus for sustainable health improvements.
